# Occupational exposure to blood-borne pathogens among health workers at a tertiary hospital in China during 2016–2022: multi-round evaluation of comprehensive intervention

**DOI:** 10.3389/fpubh.2025.1648346

**Published:** 2025-09-15

**Authors:** Wenya Shen, Min Zhang, Chuning He, Yiming Huang, Xinxin Fang, Jing Wu, Yuting Tang, Li Wang

**Affiliations:** ^1^School of Population Medicine and Public Health, Chinese Academy of Medical Sciences & Peking Union Medical College, Beijing, China; ^2^Nursing Department, The Second Nanning Peoples Hospital, Nanning, China

**Keywords:** blood-borne pathogens, occupational exposure, occupational health protection, system construction, health workers

## Abstract

**Objective:**

Occupational blood-borne pathogens exposure (OBPE) exist in all healthcare settings, and pose potential risk to health workers (HWs), comprehensive interventions are the key. This study aimed to evaluate the prevention and control measures at a tertiary hospital with 2025 beds in southern China.

**Methods:**

With the intervention of the national and international technical tools/guidance, two cross-sectional surveys were conducted in 2016 (pre-) and 2022 (post-) with the same questionnaire and methodology, respectively.

**Results:**

The incidence rate of OBPE was significantly decreased from 14.98% (2016) to 4.94% (2022) with HWs 1052 (2016) and 3080 (2022), respectively. The average number of episodes in OBPE decreased from 0.19 (2016) to 0.07 (2022) per person per year. The OBPE knowledge training rate for HWs has significantly improved with an increase by 24.30% (pre-employment) and 9.85% (on-the-job). The reporting rate of OBPE was significantly increased from 29.97% (2016) to 63.96% (2022). However, hepatitis B vaccination coverage decreased from 80.15% (2016) to 71.71% (2022), level of awareness in HBsAb status decreased from 71.32% (2016) to 58.05% (2022), HBsAb positive rate decreased from 54.80% (2016) to 39.70% (2022). The reporting rates for OBPE rose from 28.97% (2016) to 63.96% (2022), The OBPE reporting rate increased by 1.21 times. Besides, influencing factors of OBPE indicated that department, vaccination, HBsAb status, knowledge scores, working time, HBV infection status and whether report occupational health issues to the hospital were related to the occurrence of OBPE.

**Conclusion:**

Occupational health for all HWs should not be achieved once and for all, it requires a sustainable investment by systematic interventions. The valuable experiences and lessons derived here can be shared beyond the hospital in China and globally.

## 1 Introduction

Blood-borne pathogens (BBPs) are infectious microorganisms in human blood that can cause disease in humans. These pathogens include, but are not limited to, hepatitis B (HBV), hepatitis C (HCV) and human immunodeficiency virus (HIV) (1). Occupational exposure to blood-borne pathogens (OBPE) refers to incidents in which health workers (HWs), during their professional activities, are exposed to blood or other potentially infectious materials (OPIM) that contain BBPs via percutaneous injuries (e.g., a needlestick or cut with a sharp object), mucous membranes contact (e.g., eyes, nose, or mouth), or nonintact skin (e.g., exposed skin that is chapped, abraded, or afflicted with dermatitis) (2). Such exposure frequently occurs in various healthcare settings, leading to serious consequences. It not only harms the psychological and physiological health of HWs (3), but also imposes a substantial economic burden on society (4). The issue of OBPE is even more severe in developing countries (5). Sharp object injuries (SOI) are among the most representative cases. According to the publication “The World Health Report 2002*”* by the World Health Organization (WHO), among the 35 million HWs worldwide, about 3 million receive percutaneous exposures to blood-borne pathogens each year, about 40.00% of HBV and HCV infections and 2.50% of HIV infections in HWs are attributable to occupational sharps exposures (6). Factors that influence OBPE include the local socioeconomic, jobs, professional activity, work environment, and the use of medical equipment (7).

Health workers are exposed to a variety of occupational hazards in the course of their professional duties, among which OBPE constitutes one of the primary risks. A scoping review in low-and middle-income countries (LMICs) in 2021 reported that biological hazards accounted for 48%, among the studies on biological hazards, the majority (38/47) examined were OBPE (8). This study based on many years of practice following the long-term implementation of *The Guideline for Prevention and Control of Occupational Exposure to Blood Borne Pathogens* (*GBZ/T 213–2008*; referred to as the *“Guidelines”*), and integrating the (technically guided by the WHO-ILO-China) HealthWISE to the OSHMS, supported by years of sustained training efforts for standard precautions (SP) against OBPE.

The development of combating OBPE in China has mainly gone through three stages. The first stage is *Before the implementation of the Guidelines (2004–2009)* prior to the issuance of the Guidelines, OBPE was a serious and widespread issue among HWs. In 2004, the Ministry of Health of China officially included the “*Occupational Exposure Precaution Standards for Blood-Borne Pathogens”* in its national standard drafting agenda. A cross-sectional study on OBPE was conducted in a large general hospital in Beijing which revealed the total incidence and the average number of episodes exposure to BBPs was 66.3/100 HWs per year and 7.50 per person per year in the past year, respectively (9). In 2006, a survey conducted in nine hospitals across Fujian Province indicated that 71.30% of the HWs had sustained SOI during the past year, only 20.90% used safety-engineered devices during medical procedures, and 48.10% were able to correctly handle used sharps (7). These findings highlight the urgent to strengthen training and to enhance awareness and capacity for HWs on occupational health protection.

The second stage is *After the implementation of the Guidelines (2009–2012)* in order to protect HWs from occupational hazards associated with BBPs, Guidelines as one of the critical National Occupational Health Standards was officially implemented in September 2009. *The Implementation Note for Guideline* is one of the key outputs of the cooperation among the International Labor Organization (ILO) Beijing Office, the Chinese Association of STD and AIDS Prevention and Control (CASAPC) and the Chinese Center for Disease Control and Prevention (CDC). As a practical tool addressing the OSH challenge in healthcare settings, it introduces the background of the 2008 Guidelines, explains its clauses, provides technical guidance for HWs to identify occupational hazards and risks in their day to day medical practice, and encourages HWs to find solutions by themselves. In 2013, a series of comprehensive interventions were implemented at a tertiary general hospital in Shandong, which included the development of occupational health systems, updates to institutional policies and protocols, and the provision of targeted training and guidance. The post-intervention evaluation showed a marked improvement, the incidence of OBPE dropped from 81.57/100 HWs per year to 43.81/100 HWs per year, the self-reported rate increased from 2.06/100 HWs per year to 9.45/100 HWs per year (10). These findings contributed valuable practical experience and implementation recommendations.

The third stage is *After the introduction of the HealthWISE (from 2013)* the WHO/ILO technical tool HealthWISE was introduced at 2013. The Model of Hospital Initiative on Systematic Occupational (HISOH) has been applied in the pilot hospital. The core principle of the HISOH Model is the protection and maintenance of the possible highest degree of safety, health and well-being of HWs, through the establishment of a safe and healthy working environment and working conditions by means of comprehensive occupational health management system and culture (11). A national meta-analysis revealed a significant decreased in the incidence of SOI, needle stick and contaminated needle stick during 2010 to 2016, compared with 2005–2009, the incidence of SOI decreased from 84.16 to 68.23%, NSI from 80.43 to 60.39%, and contaminated NSI from 64.63 to 43.99% (12).

Occupational exposure of HWs to BBPs is preventable. Risk management should follow the hierarchy of occupational hazard controls. The first step is to eliminate hazards, followed by engineering controls, management measures, and behavioral controls, with personal protective equipment (PPE) as the last line of defense (13). Universal precaution (UP) is a general principle (14), healthcare settings have developed SP according to their own needs and practice results based on UP. Standard precautions are meant to reduce the risk of transmission of blood-borne and other pathogens from both recognized and unrecognized sources (15). Standard precautions are a fundamental set of actions HW should take as a primary infection prevention strategy, designed to limit risk of blood-borne infections, other occupational infections, and patient health care associated infections (HAI) (16). However, occupational hazards from BBPs among HWs cannot be effectively mitigated by isolated interventions, a comprehensive, multi-faceted approach is necessary to tackle complex occupational health issues. Implementation of relevant guidelines has significantly advanced Occupational Safety and Health Management System (OSHMS) in healthcare settings (17). Over the past decade, through standard implementation, application, follow-up investigation, and pilot studies, OSHMS has been established based on continuous improvement, good practice and effective model (11, 18, 19).

This study aims to evaluate the effectiveness of the current OSHMS under the national and international standard and technical tools: (i) by continuous improving the OSHMS through a six-year follow-up study on the effectiveness of the continuous intervention measures implemented in the setting; (ii) by exploring the relationship between sociodemographic and work-related factors and the experience to OBPE.

## 2 Materials and methods

### 2.1 Design

A three-phase study in a pilot hospital was conducted to assess effectiveness of the OSHMS to improve occupational health for HWs. A baseline in the first phase in October 2016 was conducted by an international questionnaire to investigate the occurrence of OBPE in the preceding year (from July 1, 2015 to June 30, 2016). The follow up investigation was conducted in 2020 to evaluate the effectiveness of initiating HealthWISE (Work Improvement in Health Services) program during COVID-19 and its impact, with results published elsewhere in 2022 (20). The third phase in June 2022 repeated the survey for further implementation of OSHMS to prevention and control OBPE, the exposure period is from July 1, 2021 to June 30, 2022.

**Figure 1:**
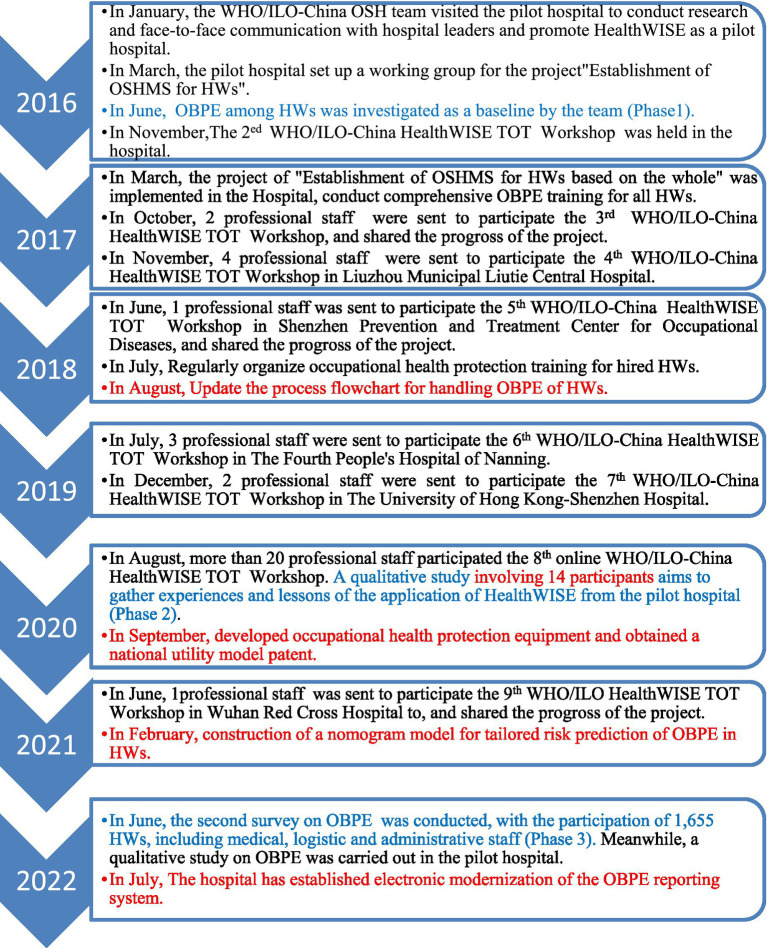
The key milestones of establishing the OSHMS and risk assessment of OBPE in the hospital from 2016 to 2022. HealthWISE: work improvement in health services; ILO and the WHO jointly developed, an international technical tool that helps health workers (HWs) to identify workplace hazards and apply low-cost solutions; a practical, participatory methodology for improving the quality of health facilities. It is a combined action and learning tool consisting of two handbooks, the Action Manual helps to initiate and sustain changes for improvement and is designed to promote learning by-doing, while the Trainers’ Guide contains guidance and tools for a training course (18). TOT, Training of Trainer.

This quasi-experimental study employed a before-and-after design with two cross-sectional surveys to evaluate the impact of OSHMS enhancements on combating OBPE outcomes. The multifaceted and sustainable intervention measures followed a structured approach, including: (a) establishment and maintaining of the Occupational Safety and Health Management System (OSHMS); (b) risk management (risk assessment and risk control) based on Occupational health standards and WHO/ILO HealthWISE tools; (c) capacity building of staff; (d) the use of safe needles; (e)the introduction of PEP measures; (f) electronic modernization of the OBPE reporting system; and (g) a comprehensive PPE upgrade program (Figure 1 for details).

### 2.2 Setting

The setting of the study is a tertiary general hospital with 3080 employees and 2025 inpatient beds, which was located in a provincial capital city within a high-prevalence HIV/AIDS region of western China in 2022. Demonstrating prioritized occupational safety and health protocols, the facility has established a comprehensive OSHMS encompassing incident recording and reporting procedure, risk assessment procedure, and immediate access to post-exposure prophylaxis (PEP) with standardized follow-up procedures. Institutional safeguards include a dedicated Occupational Exposure Prevention Committee and a Hospital Infection Control Department specifically mandated to oversee the prevention and management of BBPs transmissions among HWs (20).

### 2.3 Measurement of the study

#### 2.3.1 Study participant

The subjects of the two surveys included all HWs in the hospital, such as frontline clinical staff, administrative staff, and temporary employees. Inclusion criteria were: (1) Health workers with at least 1 year of service, including doctors, nurses, administrative staff, security, logistics workers, and outsourced staff (e.g., medical waste handlers); (2) those who provided informed consent and voluntarily participated. Exclusion criteria were: (1) staff on leave or studying outside during the survey; (2) those unwilling to participate.

In 2016, 781 of 1052 eligible HWs completed paper-based surveys (valid response rate: 74.24%). In 2022, electronic surveys yielded 1565 completed responses from 1655 eligible HWs, with 1559 valid responses (total/valid response rates: 94.56%/94.20%).

#### 2.3.2 Measurement

The study utilized the standardized “Blood-borne Pathogen Occupational Exposure Survey Questionnaire,” a validated instrument previously employed in multiple tertiary institutions across China (9, 21–23). A baseline cross-sectional survey of OBPE was initially implemented at the hospital in 2016 using the same instrument (23). The current investigation represents a follow-up cross sectional study using the same questionnaire. Trained investigators electronically distributed de-identified questionnaires via secure web-based platforms, including institutional WeChat portals, with HWs are called for participation and completed the online survey.

The questionnaire primarily collected data across four domains: (a) Demographic and occupational characteristics of participants; (b) Knowledge related to OBPE and preventive measures; (c) Incidence of OBPE during the preceding 12 months; (d) Post-exposure management practices following occupational exposures. The knowledge section comprised 10 binary-scored items (1 point/correct answer) assessing core OBPE prevention competencies. Six items demonstrating both clinical relevance (via expert consensus) and statistical discriminability (*p* < 0.05 in pre-testing) were selected for longitudinal comparison.

#### 2.3.3 Statistical analysis

The survey data were statistically analyzed using SPSS 26.0 (SPSS Inc., Chicago, IL, United States) software. Categorical variables were described using composition ratio and frequency, the data were examined at Chi-square value. Significant factors were modeled in binary logistic regression analysis to calculate ORs with CIs by using the forward stepwise (likelihood ratio) method. The data were examined at 95% *CIs*, and *p* < 0.05 was deemed statistically significant. As for dependent variable, the exposed is assigned a value of 1, while the unexposed is assigned a value of 0. It was the risk of being exposed.

#### 2.3.4 Quality control

All researchers were trained with respect to the background of the investigation. Before the study began, the study team members explained the purpose, importance, survey arrangement, and questionnaire completion methods to the study subjects in various departments. Two researchers validated the data and deleted logical errors after collecting the questionnaire. The database was then analyzed.

By cross-referencing and validating the survey data with the registry records, we performed a calibration, yielding the following adjusted incidence and reporting rates for OBPE.(1) Actual Incidence Rate of OBPE = [Number of HWs reporting exposure in the survey + Number of HWs recorded in the registry – Number of overlapping cases (matched between survey and registry)] / Total number of surveyed HWs × 100.00%. (2) Actual Reporting Rate of OBPE = Episode of exposure incidents recorded in the registry / [Episode of exposure incidents reported in the survey + Episode of exposure incidents recorded in the registry - Episode of overlapping incidents (matched between survey and registry)] × 100.00%. This formulation ensures methodological rigor by accounting for duplicate reporting and minimizing selection and recall biases. The adjusted rates provide a more accurate representation of true exposure incidence and reporting compliance. However, due to the limited content of the registration report, only the reporting and occurrence situations have been adjusted.

## 3 Results

### 3.1 Incidence of OBPE

Table 1 presents the incidence of OBPE. Compared to the baseline survey in 2016, the incidence rate (IR) and incidence density (ID) showed a decline in 2022. The incidence rate of OBPE decreased from 14.98 to 4.94% (*p* < 0.001), SIs declined from 12.29 to 4.04% (*p* < 0.001), while skin and mucous membrane exposure (SMME) dropped from 3.20 to 1.86% (*p* < 0.05). Additionally, the average number of episode exposure to BBPs showed a decline in 2022. The average number of episodes in OBPE decreased from 0.19 to 0.07 per person per year (*p* < 0.001), SPs declined from 0.14 to 0.05 per person per year (*p* < 0.001), while SMME dropped from 0.05 to 0.02 per person per year (*p* < 0.001). Overall IR and ID of OBPE decreased by 2.03 and 1.7 times, SIs decreased by 2.04 and 1.8 times, while SMME decreased by 0.72 and 1.50 times.

**Table 1:** Incidence of OBPE between 2016 and 2022 in the tertiary hospital.

Route of exposure	Baseline survey (N_b_ = 781)				Follow up survey (N_f_ = 1559)				Decrease of incidence rate (times)	Decrease of incidence density (times)
	Exposed health works (n)	Incidence rate (%)	Frequency (n)	Incidence density (No./person/year)	Exposed health works (n)	Incidence rate (%)	Frequency (n)	Incidence density (No./person/year)		
SOI	96	12.29	106	0.14	63	4.04	75	0.05	2.04***	1.8***
SMME	25	3.20	39	0.05	29	1.86	36	0.02	0.72*	1.5***
OBPE	117	14.98	145	0.19	77	4.94	111	0.07	2.03***	1.7***

*** indicates *p* < 0.001; * indicates *p* < 0.05; SOI, Sharp object injuries; SMME, skin & mucous membrane exposure; OBPE, Occupational blood-borne pathogens exposure; Incidence density, Episodes per person per year; Frequency: No. of episodes; the exposure period in baseline survey from July 1, 2015 to June 30, 2016; The exposure period in follow up survey is from July 1, 2021 to June 30, 2022.

### 3.2 Effectiveness of intervention measures against OBPE

Effectiveness of intervention measures against OBPE among HWs were assessed between the baseline (N_b_ = 781) and the follow-up (N_f_ = 1559) surveys. Statistically significant changes were observed in training, vaccination and accuracy of OBPE knowledge. Firstly, there were some good changes of improved practices, including pre-employment training rate rose from 62.74 to 87.04%, on-the-job training rate rose from 87.58 to 97.43%, OBPE-specific knowledge training rate also improved significantly from 85.28 to 92.68%, The accuracy of “reduction of unnecessary injections” rose from 70.17 to 80.18%, “PEP for HIV” rose from 95.52 to 97.43%. Secondly, there were unfavorable changes which required immediate attention, including hepatitis B vaccination coverage decreased from 80.15 to 71.71%, level of awareness in HBsAb status decreased from 71.32 to 58.05%, HBsAb positive rate decreased from 54.80 to 39.70%. The accuracy of “Whether immediate transfer from duty post after exposure” declined sharply from 62.61 to 41.24%. Thirdly, some indicators showed no statistical significance, the accuracy of “recognition of mucous membrane and nonintact skin exposure risks” remained high, with a slight decline from 99.36 to 97.97%. Some indicators remained very low which need to be paid attention urgently, despite the accuracy of “hierarchy of occupational risk control” rose from 5.12 to 5.58%, “emergency response measures after exposure” rose from 3.84 to 5.45% (Table 2 for detailed information).

**Table 2:** Changes of interventions of OBPE between 2016 and 2022 in the tertiary hospital.

Item	Baseline survey (N_b_ = 781)		Follow up survey (N_f_ = 1559)	
	n	%	n	%
Occupational health training				
Pre-employment training***	490	62.74	1357	87.04
On-the-job training***	684	87.58	1519	97.43
OBPE training***	666	85.28	1482	92.68
Immune status				
Hepatitis B vaccination status***	626	80.15	1118	71.71
Positive for HBsAb ***	428	54.80	619	39.70
Awareness in HBsAb status***	557	71.32	905	58.05
Accuracy of OBPE knowledge				
Hierarchy of occupational risk control	40	**5.12**	87	**5.58**
Immediately transfer from the post after OBPE***	489	62.61	643	41.24
Reduction of unnecessary injections***	548	70.17	1250	80.18
Emergency response measures after OBPE	30	**3.84**	85	**5.45**
PEP for HIV**	746	95.52	1519	97.43
Mucous membrane, nonintact skin exposure risk	776	99.36	915	97.97

*** indicates *p* < 0.001; ** indicates *p* < 0.01; PEP, Post-exposure prophylaxis; the exposure period in baseline survey from July 1, 2015 to June 30, 2016; the exposure period in follow up survey is from July 1, 2021 to June 30, 2022. The bold values mean the date of indicators <10% which need to be paid attention urgently.

### 3.3 Evaluation of effectiveness in protective measures against OBPE

Personal protective equipment compliance among HWs during OBPE was evaluated between baseline (N_b_ = 145) and follow up surveys (N_f_ = 87). Changes of key metrics including frequency and utilization rates for 5 PPE categories were statistical significance. Firstly, there were some improved practices, including double-layer gloves rose from 4.83 to 16.09%, gauze mask decreased from 70.34 to 9.20%, surgical masks increased markedly from 14.48 to 44.83%. Still, there were unfavorable changes. Surgical gowns declined significantly from 29.66 to 6.90%, ordinary work suit declined significantly from 71.03 to 14.94%. Finally, although there is no statistical significance, types of PPE with low wearing rate need to be paid attention urgently, despite goggles rose from 2.07 to 6.90%, face shield rose from 2.76 to 8.05%, surgical N95 respirators rose from 0.00 to 1.15%, waterproof aprons and coverall protective suit rose from 0.00 to 2.30% (Table 3 for detailed information).

**Table 3:** Changes of the use of PPE during OBPE between 2016 and 2022 in the tertiary hospital.

Types of PPE	Baseline survey (N_b_ = 145)		Follow up survey (N_f_ = 87)	
	F_b_ (n)	%	F_f_ (n)	%
Single-layer latex/plastic gloves	97	66.90	47	54.02
Double-layer latex/plastic gloves**	7	4.83	14	16.09
Goggles	3	2.07	6	6.90
Eyeglasses (not a protective item)	32	22.07	15	17.24
Face shield	4	2.76	7	8.05
Gauze mask***	102	70.34	8	9.20
Surgical mask***	21	14.48	39	44.83
Surgical N95 Respirator	0	0.00	1	1.15
Surgical gown***	43	29.66	6	6.90
Waterproof apron	0	0.00	2	2.30
General work clothes ***	103	71.03	13	14.94
Coverall protective suit	0	0.00	2	2.30

*** indicates *p* < 0.001; ** indicates *p* < 0.01; F, Frequency (No. of episodes). The exposure period in baseline survey from July 1, 2015 to June 30, 2016; the exposure period in follow up survey is from July 1, 2021 to June 30, 2022.

Post-exposure emergency practices between the baseline (N_b_ = 145) and the follow up surveys (N_f_ = 87) were compared. Changes of key metrics including frequency and response rates for 3 categories were statistical significance. There were two good changes of improved practices, including flushed with water after MME increased markedly from 56.67 to 100.00%, flushed with other solutions rose from 0.00 to 37.50%. However, disinfection after SOI declined from 97.17 to 87.04%. Although there was no statistical significance, three variables remained consistently high, washed with water after nonintact skin exposure both reached 100.00%, washed with water and expressed blood after SOI maintain high level with more than 90.00%. Some need to be paid attention urgently due to not so high level, despite flushed with physiological saline after MME rose from 60.00 to 75.00%, washed with soap or antiseptic-hand cleaners after nonintact skin exposure rose from 66.67 to 72.00%, and disinfection after nonintact skin exposure rose from 77.78 to 84.00% (Table 4 for detailed information).

**Table 4:** Changes of emergency response measures after OBPE between 2016 and 2022 in the tertiary hospital.

Frequency of response measures	Baseline survey (N_b_ = 145)		Follow up survey (N_f_ = 87)	
	F_b_ (n)	n (%)	F_f_ (n)	n (%)
SOI	106		54	
Wash with water		101 (95.28)		49 (90.74)
Wash with soap or antiseptic-hand cleaners		91 (85.85)		43 (79.63)
Express blood		100 (94.34)		49 (90.74)
Disinfection *		103 (97.17)		47 (87.04)
MME	30		8	
Flush with physiological saline		18 (60.00)		6 (75.00)
Flush with water*		17 (56.67)		8 (100.00)
Flush with other solutions**		0 (0.00)		3 (37.50)
Nonintact skin exposure	9		25	
Wash with water		9 (100.00)		25 (100.00)
Wash with soap or antiseptic-hand cleaners		6 (66.67)		18 (72.00)
Disinfection		7 (77.78)		21 (84.00)

* indicates *p* < 0.05; ** indicates *p* < 0.01; F, Frequency (No. of episodes); SOI, Sharp object injuries; MME, mucous membrane exposure; the exposure period in baseline survey from July 1, 2015 to June 30, 2016; the exposure period in follow up survey is from July 1, 2021 to June 30, 2022.

A comparative evaluation between baseline (N_b_ = 781) and follow-up (N_f_ = 1559) surveys reveals a marked improvement in the reporting of OBPE. The reporting rates for OBPE rose from 28.97 to 63.96% (*p* < 0.001), the reporting rates for SIs rose from 33.96 to 68.00% (*p* < 0.001), while the reporting rates for SMME rose from 15.38 to 55.56% (*p* < 0.001). The OBPE reporting rate increased by 1.21 times, SIs increased by 1 time, while SMME increased by 2.61 times (Table 5 for detailed information).

**Table 5:** Reporting rate of OBPE between 2016 and 2022 in the tertiary hospital.

Route of exposure	Baseline survey (N_b_ = 781)			Follow up survey (N_f_ = 1559)			Increase of reporting rate (times)
	F_b_ (n)	FR_b_ (n)	%	F_f_ (n)	FR_f_ (n)	%	
SOI***	106	36	33.96	75	51	68.00	1.00
SMME ***	39	6	15.38	36	20	55.56	2.61
OBPE***	145	42	28.97	111	71	63.96	1.21

*** indicates *p* < 0.001; SOI, Sharp object injuries; SMME, Skin & mucous membrane exposures; OBPE, Occupational blood-borne pathogens exposure; F, Frequency (No. of episodes); FR, No. of episodes reported. The exposure period in baseline survey from July 1, 2015 to June 30, 2016; the exposure period in follow up survey is from July 1, 2021 to June 30, 2022.

### 3.4 Influencing factors of OBPE in 2022

Table 6 presents the results of binary logistic regression. It indicated that department, vaccination, HBsAb status, knowledge scores, working time, HBV infection status and whether report occupational health issues to the hospital were related to the occurrence of OBPE. HWs in surgery departments were 2.71 times (*95% CI* 1.03 to 13.36) and internal medicine departments were 5.00 times (*95%CI* 1.44 to 17.40) risk of OBPE. HWs in departments that had not organized vaccination were 3.34 times (*95% CI* 1.19 to 9.37) and those unvaccinated due to personal reasons were 7.94 times (*95% CI* 1.79 to 35.21) more likely to experience OBPE. HBsAb-negative (*OR* = 2.54, *95%CI* 1.24 to 5.25) and HBsAb-positive (*OR* = 2.51, *95%CI* 1.45 to 5.84) increased the risk of suffering from OBPE. OBPE-related knowledge scores less than 6 (*OR* = 2.69, *95%CI* 1.36 to 7.94) and universal precaution knowledge scores less than 8 (*OR* = 8.56, *95%CI* 1.16 to 66.33) had an increased risk of OBPE. Working more than 8 h per day had a greater risk of OBPE (*OR* = 1.94, *95%CI* 1.12 to 3.35).

**Table 6:** Multivariate logistic regression of different forms of OBPE among 1559 HWs in 2022.

Variable	Category	Total HWs (1559)	OBPE			SOI			SMME		
			Exposed HWs (*n* = 54)	OR	95% CI	Exposed HWs (*n* = 43)	OR	95% CI	Exposed HWs (*n* = 26)	OR	95% CI
Department	Technical support	212	3	1.00	Reference	2	1.00	Reference			
	Surgery	291	14	3.71*	1.03 to13.36	14	6.15*	1.32 to 28.70			
	Internal medicine	324	20	5.00*	1.44 to17.40	16	6.25*	1.37 to 28.44			
	Gynecology and obstetrics	169	4	1.52	0.33 to7.09	4	2.71	0.46 to 15.89			
	Pediatric department	154	1	0.49	0.05 to 4.87	1	0.87	0.08 to 10.01			
	Operating	119	5	3.72	0.85 to 16.24	3	3.62	0.58 to 22.77			
	Outpatient and emergency	202	6	1.78	0.42 to 7.55	2	0.91	0.11 to 7.29			
	Others	88	1	0.91	0.09 to 9.20	1	1.51	0.13 to 18.12			
Vaccination	Vaccinated	1118	41	1.00	Reference	35	1.00	Reference	17	1.00	Reference
	Departments had not organized	66	5	3.34*	1.19 to 9.37	3	2.22	0.61 to 8.09	4	4.36*	1.44 to 13.63
	Unvaccinated due to personal reasons	15	3	7.94**	1.79 to 35.21	2	11.05**	1.77 to 68.95	1	2.60	0.27 to 24.77
	Not remember	360	5	0.60	0.23 to 1.60	3	0.44	0.13 to 1.51	4	0.76	0.24 to 2.36
HBsAb status	Unsure	654	12	1.00	Reference	7	1.00	Reference			
	Negative	286	13	2.54*	1.24 to 5.25	12	3.93**	1.46 to 10.58			
	Positive	619	29	2.51*	1.45 to 5.84	24	3.45**	1.41 to 8.45			
Knowledge scores of OBPE	≥8	281	2	1.00	Reference						
	7	549	17	1.63	0.37 to 7.16						
	≤6	104	9	2.69*	1.36 to 7.94						
Knowledge scores of UP	≥9	715	14	1.00	Reference						
	≤8	219	14	8.56*	1.16 to 63.33						
Working time	≤8 h per day	1095	30	1.00	Reference	24	1.00	Reference	13	1.00	Reference
	>8 h per day	464	24	1.94*	1.12 to 3.35	19	1.91*	1.03 to 3.51	13	2.40*	1.10 to 5.22
Report OHI to the hospital	Yes	289							10	1.00	Reference
	No	1270							16	2.58*	1.14 to 5.87
HBV infection status	HBV to negative	1218							19	1.00	Reference
	Unsure	325							5	1.05	0.37 to 2.94
	HBV to positive	16							2	6.07*	1.16 to 31.79

* indicates *p* < 0.05; ** indicates *p* < 0.01; OBPE, Occupational blood-borne pathogens exposure; HWs, Health works; SOI, Sharp object injuries; SMME, Skin & mucous membrane exposures; OHI, Occupational health issues; the exposure period in follow up survey is from July 1, 2021 to June 30, 2022.

HWs in surgery departments were 6.15 times (*95%CI* 1.32 to 28.70) and Internal medicine departments were 6.25 times (*95%CI* 1.37 to 28.44) more exposed to SOI. Those unvaccinated due to personal reasons were 11.05 times (*95%CI* 1.77 to 68.95) more likely to experience SOI. HBsAb-negative (*OR* = 3.93, *95%CI* 1.46 to 10.58) and HBsAb-positive (*OR* = 3.45, *95%CI* 1.41 to 8.45) increased the risk of suffering from SOI. Working more than 8 h per day had a greater risk of SOI (*OR* = 1.94, *95%CI* 1.12 to 3.35).

HWs who not reported occupational health issues to the hospital suffered significantly more SMME (*OR* = 2.58, *95%CI* 1.14 to 5.87). HWs in departments that had not organized vaccination were 4.36 times (*95%CI* 1.44 to 13.63) more likely to experience SMME. HWs who suffered from HBV were 3.45 times (*95%CI* 1.41 to 8.45) more exposed to SMME. Working more than 8 h per day had a greater risk of SMME (*OR* = 2.40, *95%CI* 1.10 to 5.22).

## 4 Discussion

### 4.1 A decrease in OBPE under intervention and prevention measures

Our study strongly demonstrates that the incidence rate of OBPE in this hospital has decreased from 14.98% in 2016 (24) to 11.60% in 2017 (25), and further to 4.94% in 2022. The average number of episodes in OBPE decreased from 0.19 (2016) to 0.07 (2022) per person per year. This continuous reduction indicates a positive trend of OBPE prevention effort. Compared with similar studies globally and domestically, the OBPE incidence rate documented in our study is substantially lower than rates reported elsewhere. For example, a Tanzania study conducted at three public hospitals reported 32.00% OBPE incidence rate in 2012 (26). A systematic study shows that the global prevalence of NSIs among nurses is 40.97% (27). Additionally, a cross-sectional study of 20791 nurses across 31 provincial departments in China found that 52.10% nurses had experienced OBPE in 2022 (28). A comparison of these findings suggests that remarkable achievements have been made in the construction of OSHMS.

The observed downward trend can be explained by both national-level initiatives and hospital-level interventions. In 2004, China’s Ministry of Health officially included *The Guidelines for Occupational Exposure Protection to Blood-borne Pathogens* in its drafting agenda. Subsequently, the Ministry issued directives mandating research and surveys on occupational protection against blood-borne pathogens in selected hospitals, HIV laboratories, and blood centers across all provinces, municipalities, and autonomous regions. To strengthen institutional capacity and establish model hospitals, the National Institute of Occupational Health and Poison Control of the China CDC conducted a series of intervention studies between 2009 and 2012, which resulted in the development of a quantitative assessment tool for occupational exposure protection. This tool was progressively refined through years of pilot testing. Evidence from previous reviews indicated that after the implementation of the Guideline, hospitals demonstrated clear improvements in multiple indicators, including the rate of safe sharp-use training among nurses, hepatitis B vaccination coverage, glove usage, correct wound management, and reporting of exposure incidents. These findings confirmed that the Guideline had been effectively implemented and yielded measurable benefits, though substantial room for improvement remained. The pilot results further highlighted the need for more systematic training tools that integrate occupational health perspectives.

In this national context, this present pilot project was conducted in a provincial tertiary general hospital, building upon 2010 baseline survey and guided by the Guideline. With technical support from WHO, ILO, and Chinese counterparts, the WHO/ILO HealthWISE tool-Improving Health Workers’ Working Conditions-was introduced, alongside the Hospital Initiative on Systematic Occupational Health model (HISOH model) (11). These frameworks facilitated the updating of hospital regulations and policies and enabled the implementation of comprehensive interventions, including knowledge training, group discussions, and on-site supervision. Under these measures, the hospital established an evidence-based standard precautions system and fostered a human-centered workplace culture. Guided by both practical needs and national policy directives, substantive changes were achieved-ranging from the development of intervention tools and evaluation indicators to improvements in working conditions and job design across organizational, environmental, engineering, and behavioral dimensions.

However, the COVID-19 pandemic in 2020 has contributed to a resurgence in OBPE (29), highlighting the urgent need for continued prevention efforts to minimize the occurrences. There are also studies that have shown that the frontline physicians and nurses were not disproportionately affected by COVID-19 infection, suggesting the effectiveness of a robust hygiene regimen. Despite the most intense and efficient identification of asymptomatic HWs, which may be crucial for preventing SARS-CoV-2 infections in hospitals, those with the highest patient contact were not significantly overrepresented in infection events (30, 31).

### 4.2 Effective continuous intervention measures have been taken to protect HWs from OBPE

Training has proven to be an effective intervention effectively improved HWs’ knowledge and reduced the risk of OBPE (32). Through action-oriented participatory training, it systematically enhances HWs’ knowledge and skills, resulting in a reduced risk of OBPE, a benefit that is both measurable and tangible (18). The follow up survey shows that pre-employment training rate rose from 62.74 to 87.04%, on-the-job training rate rose from 87.58 to 97.43%, OBPE-specific knowledge training rate also improved significantly from 85.28 to 92.68%. Compared with the 2010 survey in Shandong, the training rate were 40.38, 74.85, and 72.02%, respectively (21). These figures demonstrate a steady and encouraging increase in the occupational health training rates related to OBPE over the years. However, there is still room for further improvement, especially in pre-employment training. The underlying cause stems from the insufficient emphasis HWs place on occupational health protection against OBPE. Strengthening this early-stage training is crucial for building a strong foundational understanding of OBPE prevention among HWs before they engage in clinical activities. The accuracy of “hierarchy of occupational risk control” and “emergency response measures after exposure” were both about 5.00%, critical knowledge gaps remain, particularly in understanding key safety principles and emergency procedures. Strengthening education, practical training, and regular assessments are urgently needed to fully equip HWs with the skills necessary to minimize risks.

The use of PPE has been regarded as fundamental standard precautions in preventing OBPE (33). Following communication with the relevant departments of the Materials Management, the most widely allocated PPE were surgical masks (97.95%) and double-layer latex/plastic gloves (93.71%). Compared to 2016, the allocation rates of surgical masks, surgical N95 respirator, goggles, and face shield have significantly increased. Additionally, hospitals have equipped a large number of disinfection supplies and isolation gowns. In addition, it is important to acknowledge the potential influence of temporal externalities, such as COVID-19–related PPE usage and policy shifts (34), on the observed decline in OBPE rates between 2016 and 2022. During the pandemic period, strict infection prevention measures (e.g., universal PPE, social distancing, reinforced hand hygiene protocols, and revised occupational safety regulations) were implemented across healthcare institutions, which may have contributed to the reduced exposure risk. However, a survey conducted during the pandemic revealed that even though PPE, social distancing, COVID-19 guidelines (gloves, mask, and hand sanitizer), and other precautions were advised to health care professionals, the symptoms predicting a positive test for SARS-CoV-2 and its mortality were still found to be high. Moreover, despite the implementation of PPE protocols and isolation, there was no difference in risk between healthcare and nonclinical professionals (35). These findings suggest that while COVID-19–related precautions may have reinforced the overall downward trend, the reduction in OBPE cannot be fully explained by the pandemic and is more likely attributable to the multi-round interventions implemented before and during this period. Furthermore, the hospital should proactively invest in and maintain a supply of high-grade, advanced PPE (e.g., goggles or glasses with solid side shields, chin-length face shields) to ensure optimal protection for HWs. Over the past 6 years of continuous intervention and training, combined with the increased demand for PPE during the pandemic, hospitals have placed greater emphasis on the allocation and use of PPE. As a result, the personnel satisfaction level among HWs regarding PPE availability has remained relatively high which up to 96.41%. The costs associated with PPE should neither be borne by individual HWs nor deducted from departmental budgets. Clear national policies and regulations should be issued to standardize employer responsibility for PPE expenditures, ensuring that healthcare institutions provide adequate protective equipment without financial burden on staff. At the same time, mechanisms should be established to prevent both overuse and underuse of PPE, balancing cost-effectiveness with safety. Such measures would ensure equitable access to appropriate protection, safeguard occupational health, and promote sustainable resource allocation within healthcare systems.

Hepatitis B vaccination is a safe, effective, and well-established method for preventing HBV infection, offering long-term protection against the virus (36, 37). The follow up survey results show that the hepatitis B vaccination rate among HWs is 71.71%, while the positive rate of hepatitis B surface antibodies is 39.70%, and only 58.05% of HWs are aware of their immune status, all of which are lower than the levels reported in the baseline survey. By comparison, a study of 442 HWs in Egypt found that 81.7% had completed the three-dose hepatitis B vaccination regimen. The main reasons for not completing or skipping vaccination included concerns about vaccine side effects, lack of trust in vaccine efficacy, fear of injections, natural immunity (Hbs titer ≥10mIU/mL), lack of time, pre-existing health conditions, and contraindications (38). In China, the widespread administration of hepatitis B vaccine has largely been achieved through the National Immunization Program, which provides free vaccination to newborns. However, vaccine-induced immunity typically wanes over time, leaving adult workers-particularly healthcare workers exposed to hepatitis B virus-at ongoing risk. Currently, there is a lack of an occupational health immunization program that specifically addresses workplace-related risks. To better protect HWs, free access to all vaccines for vaccine-preventable diseases, including booster doses, should be systematically provided from an occupational health perspective. The fundamental issue lies in the absence of mandatory measures at the national level. The *Guidelines for Protective and Control of Occupational Exposure to Blood Borne Pathogens* are classified as recommended standards, which limits their enforceability. To address this gap, several measures are needed: (a) formally recognizing occupational hepatitis B as an occupational disease; (b) implementing mandatory hepatitis B vaccination programs for HWs; and (c) ensuring that all related vaccination costs are covered as part of occupational health and medical expenses.

Underreporting and non-reporting of occupational exposures are common across healthcare settings. A study in Shanghai revealed that respondents experienced SOI with the reporting rate was only 25.61% in 2016 (39). In 2021, a cross-sectional survey of 444 primary healthcare workers in South Africa found that 82.0% of occupational exposures went unreported. The most commonly cited reasons for non-reporting included lack of time (42.72%), perceived low risk of HIV infection from the source patient (24.70%), and concerns about confidentiality (22.50%) (40). This survey indicated that reporting rate after OBPE is 63.96%, with a reporting rate of 68.00% for SOI and 55.56% for occupational exposure to skin and mucous membranes. Compared to the 2016 survey results, these figures have shown significant improvement, reflecting refinements in the hospital’s reporting system and procedures, increased awareness of occupational protection among HWs, and notable progress in fostering a no-blame culture within the hospital.

A multifactorial logistic regression analysis of OBPE revealed that HWs in internal medicine and surgery departments face a significantly higher risk compared to those in paraclinical departments. This disparity is attributable to the inherent characteristics and demands of these clinical specialties. HWs in internal medicine and surgery typically endure heavy workloads, extended working hours, and perform a greater frequency of high-risk procedures, consequently increasing their exposure frequency to BBPs (41). Hepatitis B vaccination serves as a crucial protective measure against occupational hazards from BBPs. HWs who have not received the hepatitis B vaccine often demonstrate insufficient attention to their own occupational health protection and lack adequate occupational safety awareness, rendering them more susceptible to OBPE. HBsAb-positive HWs exhibited a higher risk of OBPE compared to those uncertain about their HBV antibody status. This paradoxical finding may arise because OBPE often prompt subsequent HBV antibody testing, follow-up monitoring, and potentially the administration of hepatitis B vaccine or immunoglobulin to exposed individuals, leading to a higher observed seropositivity rate in the exposed group. HWs with lower scores demonstrated poorer comprehension of the relevant knowledge. This indicates a lack of understanding regarding the risks of OBPE and the necessary preventive and response strategies, consequently correlating with a higher incidence of exposure incidents. Working hours serve as an independent risk factor for SOI, HWs working over 8 h per day exhibited a 1.91 times higher risk of SOI compared to those working less than 8 h. Under non-routine work schedules (e.g., extended shifts, overtime, rotational/night shifts), HWs may fail to adequately adjust their internal biological clocks (42) leading to cumulative fatigue, occupational stress, and challenges in balancing work-family responsibilities or social commitments. These conditions subsequently contribute to a decline in work performance and elevated safety risks. HWs who reported occupational hygiene issues to hospitals exhibited a 1.58 times higher risk of SMME compared to those who did not report such issues. This elevated risk may be attributed to heightened awareness of occupational protection, enhanced ability to identify workplace hazards, and increased proactive reporting of occupational health issues following initial exposure incidents.

### 4.3 Stepwise recommendations for combating OBPE at the hospital level

In this study, several gaps in occupational protection were identified: training on the safe use of sharps did not reach full coverage; some health workers did not consistently wear protective equipment that met required standards; the rate of active reporting following contaminated needlestick injuries remained suboptimal; and hepatitis B vaccination was not uniformly regulated, with considerable room for improving coverage.

To effectively address these gaps and reduce OBPE, a multi-faceted strategy is needed. First, in line with *the Law on Prevention and Control of Occupational Diseases* and *The Guideline for Occupational Exposure Protection to Blood-borne Pathogens*, hospitals should establish a preventive occupational health protection system. This includes setting up leadership and management bodies, assigning dedicated or part-time professionals, formulating relevant regulations, and ensuring the implementation of protective measures to provide healthcare workers with a safe working environment. Second, free hepatitis B vaccination should be provided for all categories of healthcare workers-including nursing interns-with follow-up to ensure vaccination success rates. Third, hospitals should establish an active surveillance and reporting system for OBPE, accompanied by clear reporting procedures and awareness campaigns to improve reporting rates. Strengthening institutional occupational health systems is therefore essential.

Beyond institutional measures, integrating psychological support-such as early screening, cognitive behavioral therapy, and peer support-into post-exposure protocols is critical for reducing trauma and fostering a blame-free reporting culture. OBPE significantly affects HWs’ psychological well-being, anxiety, depression, occupational burnout, and even post-traumatic stress disorder (PTSD) are commonly reported after OBPE (3). 41.80% of HWs in a US study reported psychological distress after NSI (43). In Canada, exposed HWs reported stigma, which was strongly correlated with the perceived severity of infection risk (44). Second, the role of hospital trade unions can also be strengthened in managing OBPE. Hospital trade unions serve not only as guardians of HWs’ rights in the context of OBPE, but also as systemic facilitators in establishing a comprehensive protection framework through institutional enhancement, resource coordination, and psychosocial support, which from prevention to rehabilitation (45). Hospital trade unions can further support this process by developing guidelines, overseeing compliance, coordinating multidisciplinary teams, and ensuring access to post-exposure prophylaxis and occupational certification. Third, establishing a standardized reporting system would allow a shift from reactive responses toward proactive prevention, improving both risk analysis and long-term monitoring (46). Beyond reducing individual harm, such systems contribute to systemic improvements in healthcare safety, ultimately mitigating long-term risks of blood-borne infections among HWs (47). Additionally, during and after exposure events, immediate emotional and logistical support-including structured follow-up and root cause analysis-should be provided to enhance individual recovery and institutional learning. Beyond addressing the harm of individual incidents, institutionalized rapid responses contribute to reshaping the safety ecosystem in healthcare settings, thereby advancing both the protection of HWs’ rights and the hospital’s risk management capabilities (48). OBPE should be managed according to SP protocols (49). Finally, empowering healthcare workers through regular training, smart protective equipment, and self-efficacy initiatives can strengthen proactive safety behaviors and foster sustainable risk reduction (50), as HWs with high self-efficacy are more likely to actively adhere to SP measures (51).

Taken together, these recommendations-grounded in the study’s findings-highlight the need for comprehensive strategies across institutional, psychological, technical, and behavioral dimensions. Such integrated measures are crucial for reducing occupational exposure to blood-borne pathogens and for cultivating a robust safety culture within healthcare settings.

### 4.4 Stepwise recommendations for combating OBPE at the national level

On the one hand, substantive changes in hospitals usually require the impetus of national policies. On the other hand, the pilot hospital selected for this study is a representative tertiary hospital, and the problems it demonstrated are common across institutions of the same level. These findings underscore the need for stepwise, nationwide recommendations for combating OBPE. Based on our results, a comprehensive strategy should be advanced at the national level, systematically integrating policy, legislation, management, technology, culture, coordination mechanisms, and social mobilization to strengthen occupational health protection for healthcare workers.

To enhance protection against OBPE, a multi-pronged national strategy is essential. First, legal and regulatory frameworks should be reinforced by including OBPE-related diseases in the list of occupational diseases, issuing specialized national regulations aligned with international standards, and promoting the widespread use of safety-engineered devices. Second, a nationwide surveillance and response network should be established, incorporating a real-time reporting platform, emergency supply reserves, and regional coordination centers to enable rapid resource deployment (52). Third, resource investment must be increased to ensure financial and full insurance coverage for all post-exposure services, support technological innovation, and integrate simulation-based training into healthcare curricula. Fourth, education and training should be strengthened through a standardized national curriculum, the inclusion of occupational safety in medical and nursing education, and public awareness campaigns. Finally, multi-sectoral and international collaboration should be promoted through cross-departmental governance, strict legal enforcement, and active engagement in global initiatives to share knowledge and align with best practices.

Together, these stepwise recommendations aim to create a systematic, responsive, and sustainable framework for reducing OBPE risks and fostering a stronger culture of occupational health and safety across China’s healthcare system.

### 4.5 Innovations and limitations of the study

This study presents a rare study in China that conducts two cross-sectional studies on OBPE within the same healthcare facility, evaluating the impact of a continuous six-year intervention program. The questionnaires and methods used in both surveys were identical, ensuring comparability, and results were validated using registry report data. The technical tool “OBPE-Questionnaire” has been effectively utilized across various health institutions in multiple provinces, including Beijing (9), Guangxi (24), Shandong (53), Fujian (54) and so on. Its validity and reliability have been rigorously verified against established standards, ensuring that the data obtained is both authentic and credible.

However, this study has certain limitations. First, although the same standardized methodology was used at both timepoints, recall bias and selection bias remain possible. The data collection relies on respondents’ recollection of events from the past 12 months, which may lead to recall bias due to memory inaccuracies or confusion. To reduce recall bias, during the data collection stage, we used the same standardized and structured questionnaires, and HWs have filled out a form every time they were exposed and conducted detailed inquiries about the OBPE events, in order to reduce the recall bias of the respondents. However, residual bias cannot be fully ruled out. Additionally, misinterpretation of questions or reluctance to disclose key information due to confidentiality concerns may introduce information bias. Since this study is a multi-year cross-sectional analysis conducted in a single tertiary general hospital, and the data is self-reported by respondents, there is a possibility of underreporting or omissions, leading to subjective bias in the results.

Second, temporal externalities such as COVID-19-related PPE usage, heightened infection control protocols, and hospital policy shifts during 2020–2022 may have acted as confounding factors in the observed decline of OBPE rates. While the reductions accelerated during the pandemic period, the downward trend was already evident in pre-pandemic years following multi-round interventions (from 14.98% in 2016 to 11.6% in 2017), suggesting that COVID-19 measures likely reinforced but did not fully account for the improvements. Nonetheless, these temporal influences should be interpreted as potential confounders.

To enhance the representativeness of future studies, it is recommended to increase the sample size and include HWs from multiple regions and different levels of healthcare facilities. Conducting multicenter longitudinal studies would provide a more comprehensive exploration of the factors influencing OBPE and allow for a more robust evaluation of intervention effectiveness.

PPE utilization rates should be interpreted by exposure type. Aggregate denominators in Table 3 reflect total incidents, while specific PPE were only relevant to subsets (e.g., face shields is typically used to prevent splash exposure). This may overestimate compliance for splash-prevention PPE. Exposure-stratified analyses for scenario-specific PPE and recommendations for procedure-specific assessments can address this limitation in future studies.

## 5 Conclusion

OBPE remains a significant public health issue, adversely impacting the physical and mental well-being of HWs. This study evaluates the impact of a six-year continuous intervention program on OBPE through two cross-sectional surveys conducted in the same health facility. A comparative analysis revealed a notable decline in occupational exposure incidence and a marked increase in reporting rates, highlighting the effectiveness of continuous interventions. Key improvements include enhanced occupational health training, pre-exposure preventive measures, and strengthened post-exposure management, reporting, and follow-up systems, Explored the influencing factors of OBPE. Strengthen occupational exposure protection against BBPs at the hospital and national levels. Psychological support following OBPE must be prioritized to safeguard the safety of HWs, strengthening the role of hospital trade unions, establishing a reporting and notification system, enhancing self-efficacy and self-empowerment in managing OBPE. Education and training should be reinforced, ensuring HWs’ awareness and compliance with safety protocols. Post-exposure handling procedures and risk assessment frameworks must be optimized for more effective management. Additionally, sustaining these efforts will enhance HWs’ health and well-being, fostering a safer and more supportive working environment, strengthen OSHMS, and contribute to overall public health protection.

## Data Availability

The raw data supporting the conclusions of this article will be made available by the authors, without undue reservation.
